# A Novel Image Encryption Approach Based on a Hyperchaotic System, Pixel-Level Filtering with Variable Kernels, and DNA-Level Diffusion

**DOI:** 10.3390/e22010005

**Published:** 2019-12-19

**Authors:** Jiang Wu, Jiayi Shi, Taiyong Li

**Affiliations:** School of Economic Information Engineering, Southwestern University of Finance and Economics, Chengdu 611130, China; wuj_t@swufe.edu.cn (J.W.); 218081202002@smail.swufe.edu.cn (J.S.)

**Keywords:** image encryption, hyperchaotic system, filtering, DNA computing, diffusion

## Abstract

With the rapid growth of image transmission and storage, image security has become a hot topic in the community of information security. Image encryption is a direct way to ensure image security. This paper presents a novel approach that uses a hyperchaotic system, Pixel-level Filtering with kernels of variable shapes and parameters, and DNA-level Diffusion, so-called PFDD, for image encryption. The PFDD totally consists of four stages. First, a hyperchaotic system is applied to generating hyperchaotic sequences for the purpose of subsequent operations. Second, dynamic filtering is performed on pixels to change the pixel values. To increase the diversity of filtering, kernels with variable shapes and parameters determined by the hyperchaotic sequences are used. Third, a global bit-level scrambling is conducted to change the values and positions of pixels simultaneously. The bit stream is then encoded into DNA-level data. Finally, a novel DNA-level diffusion scheme is proposed to further change the image values. We tested the proposed PFDD with 15 publicly accessible images with different sizes, and the results demonstrate that the PFDD is capable of achieving state-of-the-art results in terms of the evaluation criteria, indicating that the PFDD is very effective for image encryption.

## 1. Introduction

Images carry rich and direct information that is easy to perceive for the human visual system. In some specific fields, such as military, security, medical fields, and so on, it is very important to prevent image content from leaking. Therefore, image security has become a very hot research topic in the community of information security. Image encryption algorithms that change the values and/or the positions of pixels in images have been thought of as effective methods for image security. Although many popular encryption algorithms, such as DES (data encryption standard), advanced encryption standard (AES), and RSA (Rivest–Shamir–Adleman), were initially designed for block textual data, they can also be applied to encrypting images [1]. For example, AES with cipher block chaining (CBC) mode can achieve good performance in image encryption in spite of images having the apparent characteristics of bulky pixels, strong correlations, and high redundancy. Recently, chaos-based approaches have become another hot topic in the field of image encryption, since chaotic systems have many merits for encryption, such as ergodicity, unpredictability, pseudorandomness, and high sensitivity to parameters and initial values [2,3,4,5].

In chaos-based image encryption, chaotic systems are usually applied to generate chaotic sequences for changing the positions and/or values of pixels in images. Chen et al. generalized the 2D chaotic cat map to three dimensions and then applied the 3D cat map to conducting image encryption, and the results showed that the proposed scheme was fast and highly secure [2]. Pareek et al. used two Logistic maps and eight different operations to encrypt the pixels in an image, and the experiments demonstrated that the proposed approach was a secure and efficient way for image encryption [6]. Borujeni and Eshghi used a logistic map to generate a bit sequence for pseudorandom number generation in Tompinks–Paige algorithm, and the results indicated that the proposed scheme could resist any brute-force and statistical attacks [7]. Sheela et al. proposed a novel 2D Henon map with broad chaotic regime, and then used this map and sine map to confuse and diffuse images. The experimental analysis revealed the proposed scheme was advantageous over some compared traditional ones [8]. Low-dimensional chaotic systems have many advantages, such as simple form, few parameters, and easy implementation, but they are vulnerable to attack. A simple but effective solution is to use high-dimensional chaotic systems instead of low-dimensional ones. Lyapunov exponent (LE) is a poplar way to measure chaos. When a chaotic system has two or more positive LEs, it is called a hyperchaotic system, which usually has a larger key space and higher security for encryption [9,10]. Norouzi and Mirzakuchaki used two hyperchaotic systems to modify the gray-level of each pixel and crack the strong correlation among neighboring pixels in an image at the same time [11]. Zhu et al. put forward an image encryption scheme using a compound homogeneous hyperchaotic system to permute the plain image twice and then to diffuse the permutated pixels with dynamic local binary pattern operations, and the experiments demonstrated its security and effectiveness [12]. Xue et al. used a hyperchaotic system owning three positive Lyapunov exponents to encrypt the region of interest (ROI) of a color image [13]. A recently-emerged and hot research topic is to use chaotic systems and compressive sensing to encrypt and compress images simultaneously [14,15,16,17]. Some other hyperchaotic systems were also applied to image encryption [18,19,20,21,22].

As far as operations of image encryption are concerned, permutation and diffusion are among the most important ones. The former changes the positions of the data in an image, while the latter changes the values of the data. An encryption operation may involve one block of pixels, one pixel, one DNA unit (two bits), or even one bit [10,23,24,25]. The work by Xu et al. indicated that a scheme with block permutation and dynamic index based diffusion was very effective for chaotic image encryption [23]. Chaos-based S-Boxes are very popular in block encryption methods [26,27,28]. Zhang et al. proposed an image fusion encryption with a hyperchaotic system and DNA-level operations [29]. Chai et al. integrated several types of chaotic systems and DNA computing to encrypt images, showing that the proposed schemes had high security and could resist different attacks [30,31]. Khan et al. proposed a novel image encryption approach that integrated DNA computing, the intertwining logistic map, and the affine transformation for medical image encryption. The experiments demonstrated that the proposed approach was robust, efficient, and secure for medical image encryption [32]. Zhan et al. proposed a scheme with a hyperchaotic system, global bit permuting, and DNA computing (HCDNA) to improve the security and robustness of encryption [33]. In order to improve the performance of diffusion, Zhu et al. used hyperchaotic systems and ciphertext diffusion in a crisscross pattern (CDCP) to encrypt pixel-level data, and the experiments revealed the CDCP had very promising performance regarding time and diffusion [34]. Sun put forward an image encryption algorithm that used a 5D hyperchaotic system for operations on pixel-level, DNA-level and bit-level data, and both the theoretical analysis and the experimental results demonstrated that the encryption approach was secure and could resist types of attacks [35]. Zhou et al. combined a hyperchaotic system and quantum operations for bit-level image encryption [36]. To eliminate the weakness of an image encryption scheme [37], Ahmad et al. integrated discrete cosine transformation (DCT), chaotic skew tent map, and XOR operations to encrypt images. The proposed cryptosystem was capable of resisting many types of attacks and achieved very promising results in terms of several tests [38]. Very recently, Hua and Zhou have proposed a novel image cipher algorithm using block-based scrambling and image filtering (IC-BSIF), which introduced filtering, a classic operation in digital image processing, into image encryption by designing a special filter [39]. In spite of the effectiveness for image encryption, the existing filtering-based schemes usually adopt a fixed shape of filters, lacking the diversity of the filters. Hence, they may have negative impacts on encryption performance [5,10].

Motivated by the merits of hyperchaotic systems for image encryption as well as the diffusion performance by filtering and pixel-level CDCP, this paper proposes a novel scheme integrating a hyperchaotic system, pixel-level filtering with filters of different shapes, and DNA-level CDCP-like diffusion, namely, PFDD, for image encryption. PFDD consists of four stages. First, we use a 4D hyperchaotic system to generate chaotic sequences for subsequent encryption operations. Second, each pixel is filtered by a specific kernel/filter, whose shape and weights are determined by the chaotic sequences. In other words, the kernels for the pixels in an image are totally different from each other, which helps to enhance the diversity of kernels. Third, the filtered image is transformed into a bit stream, and then a global bit-level permutation is conducted on the bit stream to change the position of each bit and naturally change the values of corresponding pixels. The bit stream is then encoded into DNA-level data by rules decided by the chaotic sequences. Finally, we propose a DNA-level diffusion scheme to improve encryption performance. The main novelty of the PFDD is two-fold: (1) we propose a novel filtering operation for image encryption, which uses variable kernel shapes and kernel parameters determined by hyperchaotic sequence; and (2) we also propose a DNA-level diffusion scheme to further change the values of images.

The main contributions of this paper are as follows: (1) we use a hyperchaotic system to generate sequences for all the encryption operations; (2) kernels with variable shapes and different parameters determined by hyperchaotic sequences are used to conduct filtering to change the pixel values; (3) novel DNA-level diffusion is proposed to expand any tiny changes in a plain image to the whole cipher image; (4) pixel-level, bit-level, and DNA-level operations are used to improve the encryption effectiveness; and (5) extensive experiments demonstrate the proposed PFDD is very promising for image encryption.

The main advantages of the PFDD are three aspects: (1) permutation or diffusion is conducted with different-levels of data (pixel-level, bit-level, and DNA-level), improving the effectiveness of the PFDD; (2) a novel pixel-level filtering strategy with different kernel types and parameters determined by hyperchaotic sequences increases the diversity of kernels and hence enhances the security of the PFDD; and (3) the DNA-level diffusion is able to expand a tiny change in a plain image to the whole cipher image to resist differential attacks very well.

The rest of this paper is organized as follows. First, we briefly describe a 4D hyperchaotic system with two positive LEs, filtering operations, and DNA computing in Section 2. Then the proposed image encryption scheme that integrates the hyperchaotic system, pixel-level filtering with variable kernels, and DNA-level diffusion, is proposed in detail in Section 3. In Section 4, we display our extensive experiments on 15 testing images; the results are reported and analyzed. Finally, the paper is concluded in Section 5.

## 2. Preliminaries

### 2.1. Hyperchaotic Systems

**Figure 1:**
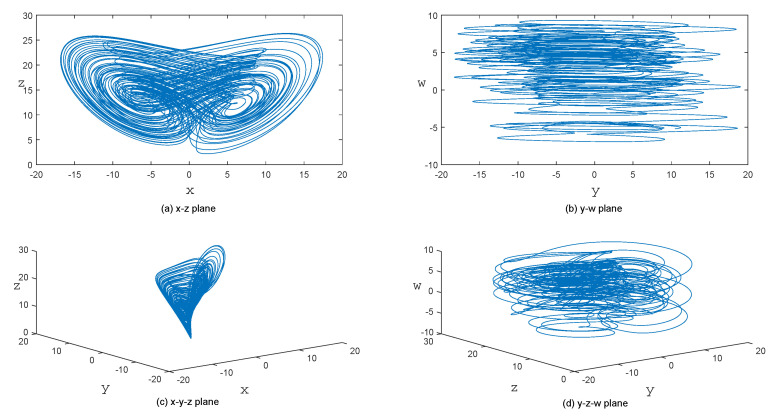
Hyperchaotic attractors of the 4D Hyperchaotic system.

Hyperchaos, first reported by Rössler [40], is usually defined as a chaotic attractor which has more than one Lyapunov exponent. Due to its advantages in security, hyperchaos is becoming more and more popular in image encryption. Recently, Gu and Gao made a 4D hyperchaotic system by adding a general linear controller to the 3D autonomous Chen’s chaotic system [41,42]. This system has two positive Lyapunov exponents and can be formulated by Equation (1):

(1) $$\left\{\begin{array}{c} \dot{x}=a\left(y-x\right)\\ \dot{y}=dx-xz+cy-w\\ \dot{z}=xy-bz\\ \dot{w}=mx+k \end{array},\right.$$

where *x*, *y*, *z*, and *w* are state variables; and *a*, *b*, *c*, *d*, *m*, and *k* are variable constants. In our work, we use the 4th-order Runge-Kutta method with a step size of h=0.001 to solve the hyperchaotic system. When the parameters (*a*, *b*, *c*, *d*, *m*, *k*) = (36, 3, 28, −16, 0.5, 0.5) and initial values ($x^{0}$, $y^{0}$, $z^{0}$, $w^{0}$) = (−1, −1, 0.3333, −5.9583), the attractors of this 4D hyperchaotic system are illustrated in Figure 1.

### 2.2. Filtering

Filtering, also known as convolution, can be used for smoothing, denoising, and sharpening images, and thus becomes essential for image processing. By applying a convolution operation between a kernel/mask/filter and an image, the pixel values of the processed image will be changed. Thus, filtering can be used for diffusing an image. However, an image filtering operation is usually irreversible, making it impossible to decrypt images. As a result, filtering cannot be directly used for diffusion in image encryption. Fortunately, Hua and Zhou proposed a new method to solve this problem: by setting the value of right-bottom position of the filtering kernel to “1,” the corresponding point in the encrypted image can be recovered [39]. In spite of the magic this technique is, there are limitations of this function, including using a kernel with a fixed shape and fixed parameters to do convolution. An ideal method should use dynamic kernel shape and variable kernel parameters for filtering.

### 2.3. DNA Computing

DNA computing could be used to solve a computational problem [43]. Different from the binary alphabets (0 and 1) in traditional computers, information is expressed by four-character genetic alphabets; i.e., A, C, G, and T for adenine, cytosine, guanine, and thymine in DNA computing, respectively. The crucial technologies of DNA computing in image encryption are encoding and decoding rules and algebraic operations. Considering the four characters of DNA alphabet, there ought to exist 4! = 24 combinations in DNA encoding. However, only eight categories of DNA combinations satisfy the DNA complementary rules, as shown in Table 1. A pixel of eight bits in a grayscale image can be encoded to four characters by using these encoding rules. For instance, a decimal value 180 can be converted to a binary value “10110100,” and further be transformed into DNA sequences “GACT” and “ACTG” by Rule 3 and Rule 8, respectively. Obviously, different encoding rules lead the identical decimal or binary value to completely different DNA sequences.

Like binary algebraic operations, DNA has its own algebraic operations, such as addition (⊕), subtraction (⊖), and XOR (⊗). Different from traditional binary operations, different DNA encoding rules can produce different results. In other words, once the encoding rule is decided, the results of DNA algebraic operations are fixed. For example, with encoding Rule 1, the results of DNA addition, subtraction, and XOR operations are listed in Table 2. These operations are usually used to change the values of DNA characters.

## 3. The Proposed Image Encryption Scheme

### 3.1. Generating Hyperchaotic Sequences

In this paper, we use the 4D hyperchaotic system described in Section 2.1 to generate the hyperchaotic sequences for encryption. Generally, the procedure is divided into three steps:

Step 1: The 4D hyperchaotic system begins to iterate to generate long enough sequences for image encryption. In the *i*-th iteration, we can obtain four state values denoted as $s^{i}=\left\{x^{i},y^{i},z^{i},w^{i}\right\}$.
Step 2: The sequences generated by the first $n_{0}$ iterations are discarded to eliminate the adverse effects.
Step 3: When the iteration completes, a hyperchaotic sequence *S* can be obtained by concatenating all the $s^{j}\left(j=1,2,\cdots ,N\right)$ as in Equation (2):
  (2) $$\begin{array}{cc} S=\{s^{1},s^{2},\cdots ,s^{N}\} & =\{x^{1},y^{1},z^{1},w^{1},\cdots ,x^{N},y^{N},z^{N},w^{N}\}\\ \; & =\{s_{1},s_{2},s_{3},s_{4},\cdots ,s_{4N-3},s_{4N-2},s_{4N-1},s_{4N}\}. \end{array}$$

Then the generated sequence *S* is further cast to an integral sequence by Equation (3):

(3) $$s_{i}=\left\lfloor \left(\left|s_{i}\right|-\left\lfloor \left|s_{i}\right|\right\rfloor \right)\times 10^{14}\right\rfloor \%256,$$

where $\left|\cdot \right|$, $\left\lfloor \cdot \right\rfloor$, and % denote the operations of absolute value, flooring, and modulo, respectively [5,9].

### 3.2. Pixel-Level Filtering with Variable Kernels

Having generated hyperchaotic sequence via Section 3.1, two sub-sequences, $S_{h}$ and $S_{w}$, can be obtained. $S_{h}$ is a 1×h vector while $S_{w}$ is a w×1 vector, so a parameter *p* can be computed by Equation (4):

(4) $$p=S_{h}\cdot I\cdot S_{w}\%256,$$

where *I* represents the plain image; *h* and *w* denote its height and width, respectively; and · is the operation of matrix multiplication. It is clear that *p* is associated with the plain image and it can be further used to change filtering kernels. In this way, different plain images will be diffused by different kernels when conducting filtering.

**Figure 2:**
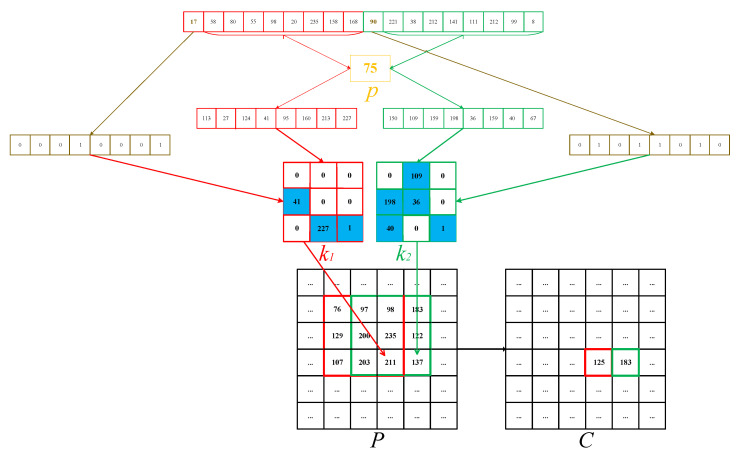
An example of multi-shape dynamic filtering.

According to the work of IC-BSIF, filtering can be used for image encryption [39]. However, it employs convolution operation to images with a kernel with a fixed shape and fixed kernel parameters values, lacking the diversity of the kernel. Very recently, Li et al. used a 1×3 or 3×1 variable kernel with different parameters to implement convolution on an image; in other words, the kernels associated with each pixel in an image for convolution are different in so-called dynamic filtering [5]. The experimental results have shown the effectiveness of dynamic filtering. Nevertheless, there still remains some room for improvement with dynamic filtering. An ideal method is to conduct filtering with variable kernel shapes and parameters, which may lead to better performance. To this end, we can use the hyperchaotic sequence to determine the shapes and parameters of the kernels. For a 3×3 kernel, since the value at the right-bottom corner is fixed to “1,” it only requires 3×3−1=8 bits to determine the kernel shapes. Fortunately, a single value in the hyperchaotic sequence is exactly an 8 bit integer, which can determine $2^{8}=256$ types of kernel shapes. For example, an 8 bit integer “0” denotes a kernel of all “0,” which means all the contents in the kernel are “0” and the shape of the kernel is blank, and hence the kernel is independent of the filtering. In contrast, an 8 bit integer “256” denotes a kernel of all “1,” implying all the values in the kernel are involved in filtering. A detailed example is shown in Figure 2. The integer “17” (“00010001” in binary) in the hyperchaotic sequence determines the shape of a 3×3 kernel, which has only three non-zero cells with blue background including the “1” in the right-bottom cell, as shown in a red border. The next eight integers first conduct bit, the XOR operation with the parameter *p* defined in Equation (4), and a new sequence containing eight integers can be obtained. Then, the new sequence is used to fill the red kernel, and we can get the kernel $k_{1}$. After that, filtering can be conducted on the 3×3 part with a red border in the plain image *P*, and then the pixel value “211” in *P* can be encrypted to “125” in the cipher image *C*. Likewise, the next nine integers in the hyperchaotic sequence can generate another kernel $k_{2}$. With this kernel and the part with a green border in *P*, the pixel “137” in *P* can be encrypted to “183” in *C*.

From this example, we can see that both the shapes and the parameters of the kernels are completely determined by the hyperchaotic sequence. The filtering operation can be applied to diffusing an image.

### 3.3. Global Bit-Level Permutation

Permutation is usually used to change the positions of pixels, and it can be further applied to permuting bit-streams. The main procedure of such an operation is as the following. First, generate a hyperchaotic sequence that has the same length as a bit-stream. Then, sort the hyperchaotic sequence to get the sorting index. Finally, rearrange the bit-stream according to the sorting index [9,33].

### 3.4. DNA-Level Diffusion

Diffusion is a frequently used way to change the pixels in images. The existing diffusion schemes are usually associated with pixel-level data or bit-level data only. Motivated by the effectiveness of CDCP [34], a pixel-level diffusion scheme, this paper proposes a DNA-level diffusion approach. In this approach, DNA addition and XOR are used to further diffuse the image since DNA algebraic operations have a property of changing the values of nucleic acids. The main idea of such DNA-level diffusion is to expand the changes in one DNA character to the whole DNA sequence. Given the length of the DNA sequence *S*, L=h×w×d/2, where *h*, *w*, and *d* denote the height, width, and depth of a plain image, respectively, and half of the *L*, H=L/2. The pseudocode of such diffusion is described as follows:

Step 1: C(1) = S(1) ⊗ (C0 ⊕ K(1)); C(H + 1) = S(H + 1) ⊗ (C(1) ⊕ K(H + 1))
Step 2: for i = 2 → H
      C(i) = S(i) ⊗ (C(H + i − 1) ⊕ K(i))
      C(H + i) = S(H + i) ⊗ (C(i) ⊕ K(H + i))
  end for
Step 3: D(1) = C(1) ⊗ (C(DL) ⊕ K(1)); D(H + 1) = C(H + 1) ⊗ (D(1) ⊕ K(H + 1))
Step 4: for i = 2 → H
      D(i) = C(i) ⊗ (D(H + i − 1) ⊕ K(i))
      D(H + i) = C(H + i) ⊗ (D(i) ⊕ K(H + i))
  end for

where ⊕ and ⊗ are DNA addition and XOR, respectively; C0 is a user-defined parameter; *K* is an auxiliary DNA-level sequence generated from the hyperchaotic system; and *D* is the obtained diffused image.

### 3.5. PFDD: The Proposed Image Encryption Approach Using a Hyperchaotic System, Pixel-Level Filtering with Variable Kernels, and DNA-Level Diffusion

**Figure 3:**
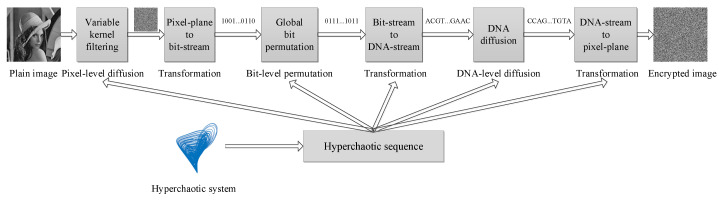
The framework of the proposed PFDD (Pixel-level Filtering with kernels of variable shapes and parameters and DNA-level Diffusion).

Due to the effectiveness of hyperchaotic systems in image encryption, permutation power of bit-level scrambling, and diffusion power of filtering and CDCP, this paper proposes a novel image encryption scheme by integrating such advantages. The proposed scheme conducts encryption on various levels, including pixel-level data, bit-level data, and DNA-level data. First, it uses a 4D hyperchaotic system with two positive LEs to generate chaotic sequences for encryption. Second, dynamic filtering operations with kernels with different shapes and parameters are conducted on pixels to diffuse the image. Third, the image is transformed into a bit stream and the global bit permutation is conducted twice. Then, the bit stream is transformed into DNA-level data. Finally, DNA-level diffusion is operated with DNA-level data, and then the DNA-level data is transformed into a pixel-level cipher image. The flowchart of the PFDD is shown in Figure 3 and the steps are described in detail as the following.

Step 1: Use initial values to generate a hyperchaotic sequence via Equations (1)–(3);
Step 2: For each pixel in the plain image, create a kernel whose shape and parameters are determined by the hyperchaotic sequence. Then, conduct a filtering operation on the pixel with the kernel. This is named pixel-level filtering with variable kernels, which results in a diffused image, as described in Section 3.2;
Step 3: Transform the diffused image into a bit stream;
Step 4: Perform the global bit permutation twice;
Step 5: Encode the bit stream into a DNA-stream. Every pair of two adjacent bits is encoded into a DNA symbol through a DNA encoding rule determined by the hyperchaotic sequence;
Step 6: Conduct DNA-level diffusion on the DNA-stream as described in Section 3.4;
Step 7: Transform the DNA-level diffused plane into a pixel plane, i.e., the cipher image.

The core of the PFDD consists of pixel-level filtering with variable kernels (Step 2), global bit permutation (Step 4), and DNA-level diffusion (Step 6). The PFDD conducts encryption in the pixel-level, bit-level, and DNA-level data, and hence it has the potential to improve encryption. The PFDD is a typical strategy of “divide and conquer”; that is, the task of image encryption is divided into several sub-tasks of encrypting different level data [44,45,46]. The decryption is the reverse of the encryption.

## 4. Experimental Results

### 4.1. Experimental Settings

In order to evaluate the performance of the proposed PFDD, some state-of-the-art encryption schemes were used for comparison, such as image encryption using pixel-level diffusion with dynamic filtering and DNA-level permutation with 3D Latin cubes (DFDLC) [10], image encryption with a hyperchaotic system and DNA computing (HCDNA) [33], CDCP [34], and IC-BSIF [39]. We set the parameters for the PFDD as follows. For the 4D hyperchaotic system, we set ($x^{0}$, $y^{0}$, $z^{0}$, $w^{0}$) = (−1, −1, 0.3333, −5.9583) and 1200 as the discard iterating time, respectively. For these comparison encryption methods’ parameters, we generally set their parameters according to the corresponding original references. We used 15 publicly-accessed, 256-level grayscale images with different sizes to test the proposed PFDD, and the sizes and names of the images are listed in Table 3. Note that Lena1024, Male2048, and Airport2048 were generated from corresponding test images with sizes of 512×512, 1024×1024, and 1024×1024 via interpolation, respectively.

All the experiments were conducted with Matlab R2017a on a PC with 64-bit Windows 10 Ultimate, 16 GB memory, and a 3.60 GHz I7 CPU.

### 4.2. Security Key Analysiss

Security keys are essential for image encryption. A large key space and high sensitivity of keys enhance the security of encryption and are capable of resisting brute-force attacks. In this subsection, we analyze those two attributes of the proposed PFDD.

#### 4.2.1. Key Space

According to the existing research, if a cryptographic system has a key space greater than $2^{100}$, it is able to resist brute-force attacks [14,47]. The initial values ($x^{0}$, $y^{0}$, $z^{0}$, $w^{0}$) for the hyperchaotic system can be used as a part of the keys of the PFDD. If the precision of each value is $10^{-15}$, the key space will be ${\left(10^{-15}\right)}^{4}=10^{-60}\approx 2^{199}$. Besides, the number of discarded iterations in generation of chaotic sequence, $n_{0}$, and the value by multiplying a chaotic sequence and the plain image (*p* in Equation (4)) can also be used as keys, enhancing the key space. Since the key space of the PFDD is much greater than $2^{100}$, it can resist brute-force attacks.

#### 4.2.2. Sensitivity to Security Keys

**Figure 4:**
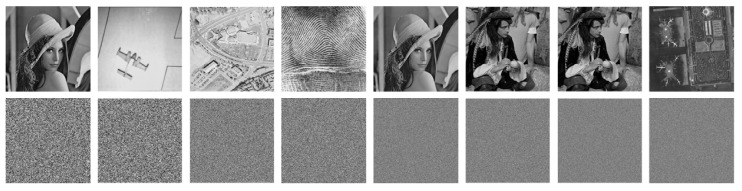
Sensitivity to security keys. From left to right, the images are Lena256, Airplane256, Aerial512, Finger512, Lena1024, Male1024, Male2048, and Airport2048.

A good and practical image encryption system should be extremely sensitive to the security keys. In other words, a tiny change with keys will lead to a completely different recovered image from the plain image. It is one of the natural characteristics of hyperchaotic systems. To verify it, we used the right security key, $K_{1}$, and a tiny change key, $K_{2}$, to decrypt some cipher images. Specifically, $K_{1}$ is ($x^{0}$, $y^{0}$, $z^{0}$, $w^{0}$) = (−1, −1, 0.3333, −5.9583), and then we added $10^{-15}$ to one of the initial value, $x^{0}$, and kept the other values were unchanged to obtain $K_{2}$; i.e., $K_{2}$ = ($x^{0}+10^{-15}$, $y^{0}$, $z^{0}$, $w^{0}$)$=(-1+10^{-15},-1,0.3333,-5.9583)$. The decrypted images with $K_{1}$ and $K_{2}$ are shown in the first and the second row in Figure 4, respectively.

It is clear that $K_{1}$ can decrypt the cipher images correctly, whereas $K_{2}$ cannot do it at all, so that the results decrypted by $K_{2}$ are random-like. The experimental results demonstrate that the sensitivity of the key of the PFDD is extremely high, which is a good attribute of an ideal image encryption system.

### 4.3. Statistical Analysis

Typical statistical analysis includes information entropy (IE) analysis, histogram analysis, and correlation analysis. The cipher images with a well-designed encryption algorithm should have evenly distributed histograms and very high entropies, and the neighboring pixels should have very weak correlations.

#### 4.3.1. Information Entropy Analysis

Information entropy, a key concept in information theory, exists to measure the degree of randomness or uncertainty in a given complex system. Typically, for a 256-level grayscale image *I*, the IE can be computed by Equation (5) [10].

(5) $$IE\left(I\right)=-\sum_{i=0}^{255}p\left(i\right)log_{2}\left(p\left(i\right)\right),$$

where p(i) indicates the probability of occurrence of the *i*-th gray level. For an image that only contains one type gray level, e.g., an all white image, the IE obtains the minimum, 0, while if all gray levels appear with the same probability, i.e., $\frac{1}{256}$, the image can achieve the highest IE, 8. A well-designed image encryption algorithm will result in an IE as close as possible to 8. The IEs of the images with the proposed PFDD and the compared algorithms are listed in Table 4.

From this table, we can see that the IEs of all plain images fall in the range of [5.3648,7.5954]—far lower than 8. In contrast, the IEs by all the encryption methods are very close or even equal to the theoretical maximum 8. More specifically, PFDD, DFDLC, HCDNA, CDCP, and IC-BSIF achieve the highest IEs with 10, 9, 4, 9, and 9 out of 15 cases, respectively. The PFDD achieved the highest IE 10 times, which is superior to the other models, indicating that the PFDD can effectively resist entropy attacks. It is worth pointing out that some entropies of the last two images are equal to 8, which shows the pixel distributions in the last two cipher images are very uniform.

#### 4.3.2. Histogram Analysis

**Figure 5:**
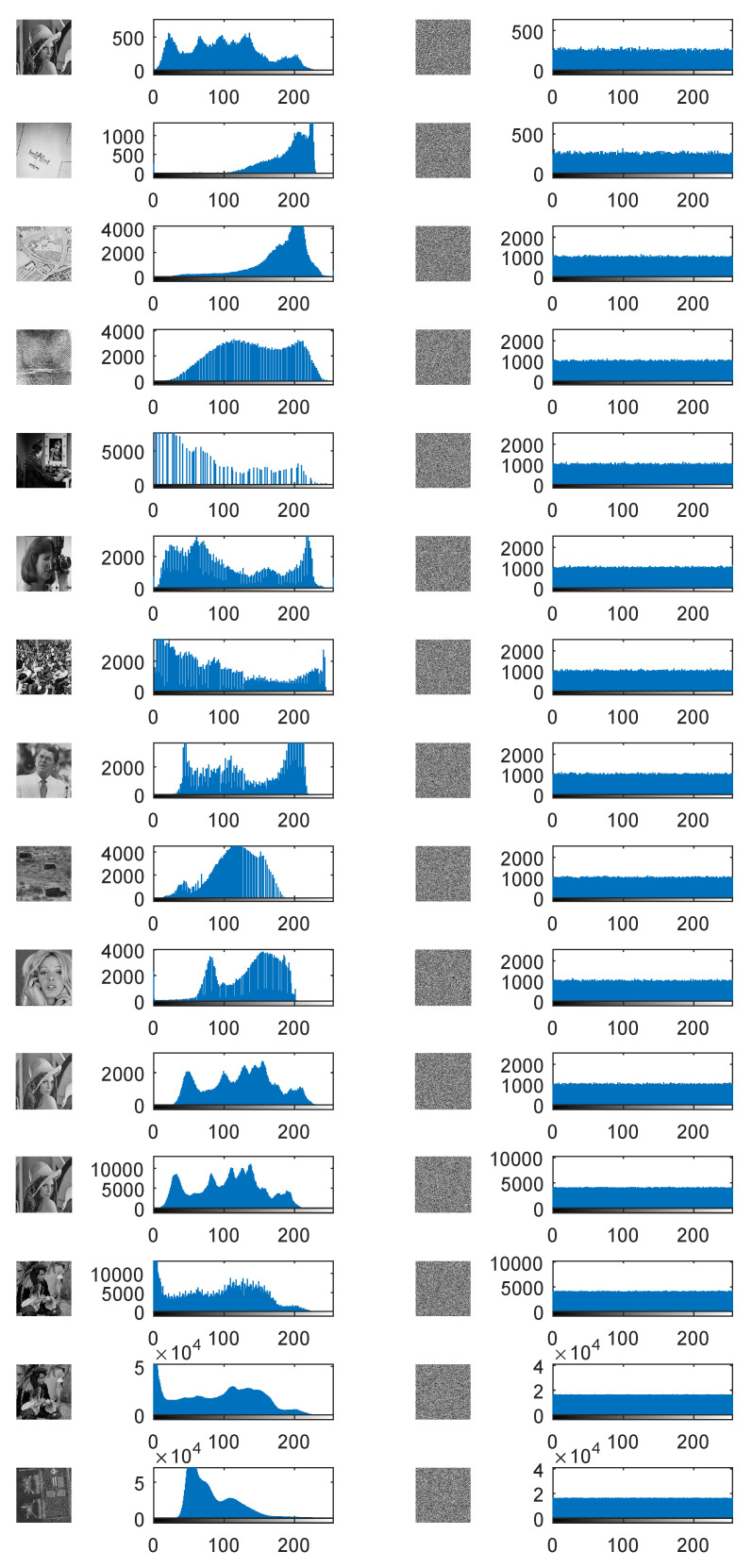
Images and histograms. From left to right, the images are plain images, the histograms of the plain images, the cipher images, and the histograms of the cipher images. In each histogram, the *x*-axis and the *y*-axis represent the pixel values and the total times the corresponding pixel occurs, respectively. From top to bottom, the names of the involved images are the same as the first column in Table 4.

A histogram is a graph that can directly reflect the distribution of pixel values in an image. The histogram of a natural image usually shows some shapes with mountains and valleys, whereas that of a cipher image by an ideal encryption algorithm should be nearly uniformly distributed to avoid histogram attacks. The images and the corresponding histograms are shown in Figure 5.

It can be found that all the histograms of plain images are very different. For example, Lena with different sizes, Finger512, Martha512, Crowd512, Male1024, and Male2048 have a wide range of grayscale values, while Airplane256, Trucks512, Woman512, and Airport2048 have a narrow one. At the same time, the different shapes of the histograms mean that the distributions of the plain images are totally different. However, when we investigate the cipher images, we can find that they are all random-like, even for the plain images with narrow pixel ranges. The histograms of all the encrypted images are so flat that they are very close to uniform distributions, showing that the proposed PFDD exhibits ideal performance regarding the histogram distribution. In particular, the tops of all bars in the histograms of cipher images with large size (the last five images) seem like horizontal lines, indicating the pixels distributes more uniformly in the cipher images with large sizes than those with small sizes.

#### 4.3.3. Correlation Analysis

Correlation reflects the relevance between two neighboring pixels in an image. Generally speaking, the correlation in a natural image is high because any two neighboring pixels are very similar, which is probably utilized to crack the image. Therefore, a practical encryption scheme should decrease such a correlation to a very low level. The correlation coefficient γ between a sequence of pixels *x* and the sequence of its neighboring pixels *y* in an image can be formulated by Equation (6) [10].

(6) $$\begin{array}{cc} E(x) & =\frac{1}{L}\sum_{i=1}^{L}x_{i},\\ D(x) & =\frac{1}{L}\sum_{i=1}^{L}{\left(x_{i}-E\left(x\right)\right)}^{2},\\ \rho (x,y) & =\frac{1}{L}\sum_{i=1}^{L}\left(x_{i}-D\left(x\right)\right)\left(y_{i}-D\left(y\right)\right),\\ \gamma & =\frac{\rho (x,y)}{\sqrt{D(x)D(y)}}, \end{array}$$

where *L* is the length of the sequence of *x*; E(x) and D(x) denote the mathematical expectation and the standard deviation of *x*, respectively; and ρ(x,y) is the covariance of the two given sequences: *x* and *y*.

For each plain image and each cipher image, we calculate the correlation coefficients in the horizontal, vertical, and diagonal directions, represented by $\gamma_{h}$, $\gamma_{v}$, and $\gamma_{d}$, respectively. Since all the pixels in an image are involved, we can think of the correlation from a global perspective. The results are listed in Table 5. We can see that the correlation coefficients of the plain images are in the range of [0.8003,0.9899], which is close to 1, confirming the strong correlation existing in natural images. However, such a strong correlation is destroyed drastically by the encryption methods. We can also find that all the correlation coefficients of the cipher images are very close to 0, showing that there is almost no correlation in the encrypted images. A typical example is the image of Martha. It has the highest correlation in the vertical direction, i.e., $\gamma_{v}=0.9899$. With the encryption schemes, however, the absolute values of $\gamma_{v}$ are less than 0.002, demonstrating the strong correlation in plain Martha has almost been completely broken. The PFDD achieves the lowest correlation coefficients in 11 out of 45 cases and all the correlation coefficients are close to 0. The results indicate that the PFDD can obtain correlation coefficients comparable to the competitive methods.

All the information entropy analysis, histogram analysis, and the correlation analysis demonstrate that the proposed PFDD can effectively resist statistical attacks.

### 4.4. Analysis of Resisting Differential Attacks

As a type of cryptanalysis, differential attacks aim to analyze how a tiny change in a plain image affects the corresponding cipher image. To defend differential attacks, a good encryption scheme should ensure that any tiny changes in the plain image are able to produce a completely different cipher image.

To measure the ability of resisting differential attacks of encryption schemes, the unified average changing intensity, UACI for short, and the number of pixels change rate, NPCR for short, are two very popular indices, as defined by Equations (7) and (8), respectively [48].

(7) $$UACI=\frac{\sum_{i=1}^{W}\sum_{j=1}^{H}\left|C_{1}\left(i,j\right)-C_{2}\left(i,j\right)\right|}{255WH}\times 100\%,$$

(8) $$NPCR=\frac{\sum_{i=1}^{W}\sum_{j=1}^{H}\delta \left(i,j\right)}{WH}\times 100\%,$$

where $C_{1}$ and $C_{2}$ are two cipher images, whose width and height are *W* and *H*, respectively, and δ(i,j) is an indicator to judge whether the two pixel values at the position of (i,j) in $C_{1}$ and $C_{2}$ are identical, which is defined as Equation (9).

(9) $$\delta \left(i,j\right)=\left\{\begin{array}{cc} 0, & C_{1}\left(i,j\right)=C_{2}\left(i,j\right)\\ 1, & C_{1}\left(i,j\right)\neq C_{2}\left(i,j\right) \end{array}.\right.$$

According to [48], for a given 256×256 8 bit gray image and a significance level α=0.05, if the UACI falls into the interval of $\left(U_{0.05}^{*l1},U_{0.05}^{*u1}\right)=\left(33.2824\%,33.6447\%\right)$, and the NPCR is greater than $N_{0.05}^{*1}=99.5693\%$, it is said that the corresponding method passes the UACI and the NPCR test at α=0.05, respectively. Likewise, if the UACI falls into $\left(U_{0.05}^{*l2},U_{0.05}^{*u2}\right)=\left(33.3730\%,33.5541\%\right)$, $\left(U_{0.05}^{*l3},U_{0.05}^{*u3}\right)=\left(33.4183\%,33.5088\%\right)$, and $\left(U_{0.05}^{*l4},U_{0.05}^{*u4}\right)=\left(33.4409\%,33.4862\%\right)$ for an 8 bit gray image of 512×512, 1024×1024, and 2048×2048, respectively, the encryption scheme also passes the UACI test. If the NPCR is greater than $N_{0.05}^{*2}=99.5893\%$, and $N_{0.05}^{*3}=99.5994\%$, $N_{0.05}^{*4}=99.6044\%$ for an 8-bit gray image of these sizes, the encryption scheme is said to pass the NPCR test.

To compute the UACI and the NPCR once, we add one to a randomly selected pixel. The computation is repeated 10 times, and the mean UACI and NPCR are listed in Table 6 and Table 7, respectively. The values that passed corresponding tests are shown in bold. From Table 6, we can see that all the UACI by PFDD, DFDLC, CDCP, and IC-BSIF fell in the specified intervals $\left(U_{0.05}^{*l1},U_{0.05}^{*u1}\right)$, $\left(U_{0.05}^{*l2},U_{0.05}^{*u2}\right)$, $\left(U_{0.05}^{*l3},U_{0.05}^{*u3}\right)$, and $\left(U_{0.05}^{*l4},U_{0.05}^{*u4}\right)$, showing they can pass the UACI test for images with all sizes of the testing images. It is worth pointing out that the PFDD achieved the highest UACI values in seven out of 15 cases. The HCDNA obtains so poor UACI that none of the image with HCDNA can pass the UACI test. As far as the NPCR is concerned, we found that PFDD, DFDLC, and IC-BSIF can pass the test. In contrast, CDCP passes the test in eight out of 15 cases, and once again, none of the images with HCDNA can pass it. The possible reason is that the encryption schemes with filtering operations (PFDD, DFDLC, and IC-BSIF) are capable of improving the performance of diffusion.

The analysis indicates that the PFDD can pass the UACI and the NPCR tests for all the experimental images, and hence it can resist differential attacks.

### 4.5. Plaintext and Ciphertext Attack Analysis

For a system of image encryption, there are four typical types of attacks; i.e., ciphertext only, chosen ciphertext, known plaintext, and chosen plaintext attacks. Among these attacks, the chosen plaintext attack is known as the most powerful one. If a cryptosystem can withstand it, it is said to have the ability to resist against other types of attacks [49].

From the aforementioned analysis, it is known that any tiny changes (even a bit) in the plain image will produce a totally different cipher image, so the proposed PFDD can resist differential attacks, which is a typically chosen plain text attack. Besides, the security keys include a value (*p* in Equation (4)) which is related to the plain image. Therefore, different plain images can generate different security keys and then obtain different results of permutation and diffusion. The cipher images by the proposed PFDD are all noise-like and all the corresponding histograms are very close to uniform distributions, further enhancing the security. In a word, the proposed PFDD highly depends on the content of the plain image, and it can resist against plaintext and ciphertext attacks.

### 4.6. Running Time and Results on Large Images

Encryption speed is another index to evaluate approaches of image encryption. Since the speed is not related to the content but to the sizes of images, we report the running time of the proposed PFDD and the compared approaches with four different types of sizes, as shown in Table 8. It can be seen that with the increase of image sizes, the running times of all the encryption approaches increase. Among the approaches, CDCP ranks first in all cases because of the simplicity of its operations, and is followed by IC-BSIF. The proposed PFDD ranks third in all cases. Since the main operations of PFDD include filtering and DNA-diffusion, its speed slightly underperforms against IC-BSIF, which conducts encryption mainly via filtering operations. DFDLC and HCDNA rank fourth and last regarding running time, respectively. Note that the running time of HCDNA is extremely high, and the possible reason is that it uses encoding/decoding rules and DNA algebraic rules directly for each operation. In the proposed PFDD, we use lookup tables instead of the rules directly for DNA encoding and DNA operations, so the running time of the PFDD is much less than that of HCDNA. Another interesting finding from this table is that the running time of all the encryption is linear with the image size. Therefore, for an encryption approach, we may estimate the running time for an image with a specific size.

A good image encryption approach should process images of different sizes well. Since the PFDD treats each unit of images (bit, DNA, and pixel) equally, there is no obvious relationship between the effectiveness of encryption and image size. In other words, the PFDD can handle images of different sizes very well. This has been demonstrated by the aforementioned analysis and discussion in terms of entropy, correlation, histogram, UACI, and NPCR. Just like a coin has two sides, the processing strategy of the PFDD limits the speed because it has to conduct filtering on the pixels one by one. Therefore, although the PFDD can achieve good encryption results for large images, it will take a lot of running time to encrypt them, and hence the time efficiency is at an intermediate level. This might be a limitation of the proposed PFDD.

## 5. Conclusions

Image encryption is very important for information security. This paper proposed a novel and effective image encryption scheme integrating a 4D hyperchaotic system, pixel-level filtering with variable kernels, and DNA-level diffusion, namely, PFDD, for image encryption. In addition, a global bit-level scrambling operation was introduced to change the position of each single bit. The advantages of the PFDD come from three aspects: (1) it performs encryption with not only pixel-data and DNA-level data, but also bit-level data; (2) the filtering kernels with different shapes and different parameters are used to enhance the diversity of the kernels, and hence improve the performance of diffusion; and (3) a DNA-level diffusion algorithm is proposed to further enhance the diffusion. We conducted extensive experiments to verify the proposed PFDD, and the results showed that the PFDD has reliable security keys and is capable of resisting types of attacks. In the future, we will extend the PFDD to color image encryption. Besides that, we will study how to improve the efficiency of the PFDD.

## Figures and Tables

**Table 1:** The encoding and decoding rules of DNA computing.

RULE	Rule 1	Rule 2	Rule 3	Rule 4	Rule 5	Rule 6	Rule 7	Rule 8
00	A	T	T	A	C	G	C	G
01	C	G	C	G	A	A	T	T
10	G	C	G	C	T	T	A	A
11	T	A	A	T	G	C	G	C

**Table 2:** DNA algebraic addition (⊕), subtraction (⊖), and XOR (⊗) operations.

⊕	A	C	G	T		⊖	A	C	G	T		⊗	A	C	G	T
A	A	C	G	T		A	A	T	G	C		A	A	C	G	T
C	C	G	T	A		C	C	A	T	G		C	C	A	T	G
G	G	T	A	C		G	G	C	A	T		G	G	T	A	C
T	T	A	C	G		T	T	G	C	A		T	T	G	C	A

**Table 3:** Testing images.

Image	Size (h×w)	Image	Size (h×w)	Image	Size (h×w)
Lena256	256×256	Airplane256	256×256	Aerial512	512×512
Finger512	512×512	Clown512	512×512	Martha512	512×512
Crowd512	512×512	Reagan512	512×512	Trucks512	512×512
Woman512	512×512	Lena512	512×512	Lena1024	1024×1024
Male1024	1024×1024	Male2048	2048×2048	Airport2048	2048×2048

**Table 4:** The information entropies (IEs) of the test images.

Image	Input	Cipher Images				
		PFDD	DFDLC [10]	HCDNA [33]	CDCP [34]	IC-BSIF [39]
Lena256	7.5954	**7.9973**	7.9971	7.9965	7.9966	7.9972
Airplane256	6.4523	7.9972	7.9969	7.9962	**7.9973**	**7.9973**
Aerial512	6.9940	**7.9993**	**7.9993**	7.9985	**7.9993**	**7.9993**
Finger512	6.7279	**7.9993**	**7.9993**	7.9990	7.9992	7.9992
Clown512	5.3684	7.9992	7.9993	7.9892	7.9992	**7.9994**
Martha512	7.5222	**7.9993**	**7.9993**	7.9991	**7.9993**	**7.9993**
Crowd512	7.4842	7.9992	7.9993	7.9946	**7.9994**	7.9993
Reagan512	7.1923	**7.9993**	**7.9993**	**7.9993**	**7.9993**	7.9992
Trucks512	6.5632	**7.9994**	**7.9994**	**7.9994**	7.9993	7.9993
Woman512	6.9542	7.9992	7.9992	**7.9993**	7.9992	**7.9993**
Lena512	7.4455	7.9993	7.9993	**7.9994**	7.9993	7.9993
Lena1024	7.4439	**7.9998**	**7.9998**	7.9991	**7.9998**	**7.9998**
Male1024	7.5237	**7.9998**	**7.9998**	7.9940	**7.9998**	**7.9998**
Male2048	7.5369	**8.0000**	**8.0000**	7.9935	**8.0000**	**8.0000**
Airport2048	6.8106	**8.0000**	**8.0000**	7.9994	**8.0000**	**8.0000**

**Table 5:** The correlation coefficients γ of the test images.

Image	γ	Input	Cipher Images				
			PFDD	DFDLC [10]	HCDNA [33]	CDCP [34]	IC-BSIF [39]
	$\gamma_{h}$	0.9144	−0.0014	0.0045	−0.0042	0.0041	**−0.0004**
Lena256	$\gamma_{v}$	0.9545	0.0028	0.0012	−0.0011	**0.0004**	−0.0020
	$\gamma_{d}$	0.9098	0.0066	0.0001	0.0029	**−0.0000**	0.0028
	$\gamma_{h}$	0.9562	0.0080	−0.0038	−0.0040	−0.0027	**0.0009**
Airplane256	$\gamma_{v}$	0.8742	−0.0104	0.0004	−0.0007	**0.0001**	−0.0036
	$\gamma_{d}$	0.8995	**−0.0000**	−0.0019	0.0003	0.0022	−0.0022
	$\gamma_{h}$	0.8993	−0.0024	−0.0009	**0.0007**	0.0009	−0.0014
Aerial512	$\gamma_{v}$	0.8549	−0.0011	0.0021	−0.0011	**−0.0009**	0.0014
	$\gamma_{d}$	0.8003	**0.0003**	0.0005	0.0021	0.0010	−0.0011
	$\gamma_{h}$	0.9343	0.0002	**−0.0001**	0.0007	−0.0023	−0.0026
Finger512	$\gamma_{v}$	0.9168	0.0013	**0.0002**	0.0029	−0.0032	−0.0030
	$\gamma_{d}$	0.8664	**−0.0007**	0.0017	−0.0022	−0.0010	0.0011
	$\gamma_{h}$	0.9763	−0.0018	−0.0026	**0.0001**	0.0019	0.0022
Clown512	$\gamma_{v}$	0.9888	0.0009	**−0.0004**	0.0020	−0.0033	0.0012
	$\gamma_{d}$	0.9699	**−0.0002**	**0.0002**	0.0010	−0.0008	−0.0015
	$\gamma_{h}$	0.9864	0.0014	0.0020	0.0002	−0.0009	**−0.0001**
Martha512	$\gamma_{v}$	0.9899	−0.0017	0.0008	**−0.0003**	**0.0003**	−0.0013
	$\gamma_{d}$	0.9815	−0.0015	−0.0004	0.0014	**−0.0003**	−0.0030
	$\gamma_{h}$	0.9059	0.0021	**−0.0003**	−0.0004	0.0019	−0.0013
Crowd512	$\gamma_{v}$	0.9047	**0.0001**	0.0014	−0.0029	−0.0005	0.0003
	$\gamma_{d}$	0.8525	−0.0018	−0.0022	0.0017	**−0.0007**	0.0012
	$\gamma_{h}$	0.9668	−0.0031	**0.0003**	−0.0017	**0.0003**	0.0015
Reagan512	$\gamma_{v}$	0.9757	0.0010	0.0003	−0.0007	0.0035	**−0.0002**
	$\gamma_{d}$	0.9573	**0.0005**	0.0008	0.0013	0.0022	0.0023
	$\gamma_{h}$	0.9408	−0.0016	−0.0034	**−0.0013**	0.0014	0.0028
Trucks512	$\gamma_{v}$	0.9110	0.0017	−0.0021	**−0.0003**	−0.0023	−0.0019
	$\gamma_{d}$	0.8906	−0.0008	**0.0000**	0.0001	−0.0029	−0.0007
	$\gamma_{h}$	0.9250	0.0028	**0.0002**	−0.0032	0.0008	−0.0004
Woman512	$\gamma_{v}$	0.9570	−0.0013	−0.0015	0.0008	−0.0020	**0.0003**
	$\gamma_{d}$	0.9217	0.0011	0.0014	0.0030	**0.0003**	**−0.0003**
	$\gamma_{h}$	0.9691	0.0013	0.0023	−0.0015	**−0.0004**	0.0023
Lena512	$\gamma_{v}$	0.9841	0.0021	**0.0009**	−0.0020	0.0028	**0.0009**
	$\gamma_{d}$	0.9639	0.0013	**0.0008**	0.0024	0.0016	**0.0008**
	$\gamma_{h}$	0.9918	**0.0007**	0.0008	−0.0012	0.0015	0.0008
Lena1024	$\gamma_{v}$	0.9962	−0.0007	**−0.0003**	−0.0020	−0.0012	**−0.0003**
	$\gamma_{d}$	0.9902	−0.0004	**0.0001**	0.0001	−0.0005	**0.0001**
	$\gamma_{h}$	0.9769	−0.0012	**−0.0001**	−0.0003	−0.0005	**−0.0001**
Male1024	$\gamma_{v}$	0.9804	**0.0008**	0.0014	0.0011	0.0009	0.0014
	$\gamma_{d}$	0.9669	0.0009	0.0008	**−0.0002**	0.0006	0.0008
	$\gamma_{h}$	0.9942	0.0014	**0.0001**	**0.0001**	0.0002	**0.0001**
Male2048	$\gamma_{v}$	0.9950	**−0.0002**	**0.0002**	−0.0004	**−0.0002**	**0.0002**
	$\gamma_{d}$	0.9905	**0.0002**	0.0004	−0.0003	**0.0002**	0.0004
	$\gamma_{h}$	0.9781	0.0009	0.0010	−0.0003	**0.0001**	0.0010
Airport2048	$\gamma_{v}$	0.9764	−0.0004	**0.0001**	−0.0007	−0.0003	**0.0001**
	$\gamma_{d}$	0.9581	**−0.0002**	**0.0002**	−0.0006	**−0.0002**	**0.0002**

**Table 6:** The average unified average changing intensities (UACI, in precentages) of running the schemes 10 times.

Image	PFDD	DFDLC [10]	HCDNA [33]	CDCP [34]	IC-BSIF [39]
Lena256	**33.4440**	**33.4741**	18.7430	**33.4862**	**33.4200**
Airplane256	**33.4620**	**33.4367**	20.3208	**33.5691**	**33.4330**
Aerial512	**33.4745**	**33.4471**	22.1490	**33.4430**	**33.4575**
Finger512	**33.4711**	**33.4095**	13.0616	**33.4836**	**33.4601**
Clown512	**33.4742**	**33.4437**	26.4164	**33.4142**	**33.4787**
Martha512	**33.4810**	**33.4748**	22.0456	**33.4501**	**33.4810**
Crowd512	**33.4718**	**33.4624**	21.2259	**33.4466**	**33.4612**
Reagan512	**33.4267**	**33.4657**	13.9140	**33.4909**	**33.5007**
Trucks512	**33.4885**	**33.4700**	25.9466	**33.4382**	**33.4385**
Woman512	**33.5120**	**33.4505**	21.6499	**33.4779**	**33.4719**
Lena512	**33.4840**	**33.4363**	26.4423	**33.4275**	**33.4568**
Lena1024	**33.4776**	**33.4674**	31.1754	**33.4320**	**33.4630**
Male1024	**33.4459**	**33.4536**	30.3316	**33.4876**	**33.4475**
Male2048	**33.4587**	**33.4641**	23.7265	**33.4629**	**33.4683**
Airport2048	**33.4556**	**33.4550**	29.0287	**33.4661**	**33.4590**

**Table 7:** The average number of pixels change rates (NPCRs (%)) of running the schemes 10 times.

Image	PFDD	DFDLC [10]	HCDNA [33]	CDCP [34]	IC-BSIF [39]
Lena256	**99.6124**	**99.6202**	46.0794	**100.0000**	**99.6045**
Airplane256	**99.6260**	**99.6155**	47.1913	**100.0000**	**99.5866**
Aerial512	**99.6101**	**99.6130**	55.1017	99.5516	**99.6142**
Finger512	**99.5956**	**99.6077**	30.8046	**99.6445**	**99.6118**
Clown512	**99.6141**	**99.6107**	60.8291	99.4683	**99.6124**
Martha512	**99.6112**	**99.6056**	54.7043	**99.6180**	**99.6130**
Crowd512	**99.6112**	**99.6066**	59.4704	99.5816	**99.6156**
Reagan512	**99.6111**	**99.6054**	35.9236	**99.5967**	**99.6089**
Trucks512	**99.6112**	**99.6121**	67.8079	**99.6015**	**99.6055**
Woman512	**99.6120**	**99.6133**	58.5091	99.5684	**99.6168**
Lena512	**99.6062**	**99.6140**	94.1631	99.2096	**99.6173**
Lena1024	**99.6075**	**99.6100**	78.9105	99.2248	**99.6100**
Male1024	**99.6113**	**99.6107**	78.9105	99.2470	**99.6084**
Male2048	**99.6104**	**99.6092**	87.1085	**100.0000**	**99.6099**
Airport2048	**99.6077**	**99.6089**	87.1085	**100.0000**	**99.6088**

**Table 8:** Running time (in seconds).

Image Size	PFDD	DFDLC [10]	HCDNA [33]	CDCP [34]	IC-BSIF [39]
256 × 256	0.9802	2.4491	8.2463	**0.2274**	0.6879
512 × 512	3.8264	7.6971	30.4855	**0.7035**	3.1478
1024 × 1024	14.4212	36.2733	123.9747	**2.7289**	9.9495
2048 × 2048	56.4122	127.8365	494.1457	**10.6423**	39.881

## References

[1] Singh G. (2013). A study of encryption algorithms (RSA, DES, 3DES and AES) for information security. Int. J. Comput. Appl..

[2] Chen G., Mao Y., Chui C.K. (2004). A symmetric image encryption scheme based on 3D chaotic cat maps. Chaos Solitons Fractals.

[3] Li X., Li T., Wu J., Xie Z., Shi J. (2019). Joint image compression and encryption based on sparse Bayesian learning and bit-level 3D Arnold cat maps. PLoS ONE.

[4] Zhou S., Zhang Q., Wei X., Zhou C. (2010). A Summarization on Image Encryption. IETE Tech. Rev..

[5] Li X., Xie Z., Wu J., Li T. (2019). Image Encryption Based on Dynamic Filtering and Bit Cuboid Operations. Complexity.

[6] Pareek N.K., Patidar V., Sud K.K. (2006). Image encryption using chaotic logistic map. Image Vis. Comput..

[7] Borujeni S.E., Eshghi M. (2009). Chaotic Image Encryption Design Using Tompkins-Paige Algorithm. Math. Probl. Eng..

[8] Sheela S.J., Suresh K.V., Tandur D. (2018). Image encryption based on modified Henon map using hybrid chaotic shift transform. Multimed. Tools Appl..

[9] Li T., Yang M., Wu J., Jing X. (2017). A Novel Image Encryption Algorithm Based on a Fractional-Order Hyperchaotic System and DNA Computing. Complexity.

[10] Li T., Shi J., Li X., Wu J., Pan F. (2019). Image Encryption Based on Pixel-Level Diffusion with Dynamic Filtering and DNA-Level Permutation with 3D Latin Cubes. Entropy.

[11] Norouzi B., Mirzakuchaki S. (2014). A fast color image encryption algorithm based on hyper-chaotic systems. Nonlinear Dyn..

[12] Zhu H., Zhang X., Yu H., Zhao C., Zhu Z. (2017). An image encryption algorithm based on compound homogeneous hyper-chaotic system. Nonlinear Dyn..

[13] Xue H.W., Du J., Li S.L., Ma W.J. (2018). Region of interest encryption for color images based on a hyperchaotic system with three positive Lyapunov exponets. Opt. Laser Technol..

[14] Chai X., Zheng X., Gan Z., Han D., Chen Y. (2018). An image encryption algorithm based on chaotic system and compressive sensing. Signal Process..

[15] Gong L., Qiu K., Deng C., Zhou N. (2019). An optical image compression and encryption scheme based on compressive sensing and RSA algorithm. Opt. Lasers Eng..

[16] Zhou N., Jiang H., Gong L., Xie X. (2018). Double-image compression and encryption algorithm based on co-sparse representation and random pixel exchanging. Opt. Lasers Eng..

[17] Zhu S., Zhu C. (2019). A new image compression-encryption scheme based on compressive sensing and cyclic shift. Multimed. Tools Appl..

[18] Tong X., Liu Y., Zhang M., Xu H., Wang Z. (2015). An Image Encryption Scheme Based on Hyperchaotic Rabinovich and Exponential Chaos Maps. Entropy.

[19] Wang Z., Min F., Wang E. (2016). A new hyperchaotic circuit with two memristors and its application in image encryption. AIP Adv..

[20] Zhang J., Hou D., Ren H. (2016). Image Encryption Algorithm Based on Dynamic DNA Coding and Chen’s Hyperchaotic System. Math. Probl. Eng..

[21] Yu S., Zhou N., Gong L., Nie Z. (2020). Optical image encryption algorithm based on phase-truncated short-time fractional Fourier transform and hyper-chaotic system. Opt. Lasers Eng..

[22] Sun S., Guo Y., Wu R. (2019). A Novel Image Encryption Scheme Based on 7D Hyperchaotic System and Row-column Simultaneous Swapping. IEEE Access.

[23] Xu L., Gou X., Li Z., Li J. (2017). A novel chaotic image encryption algorithm using block scrambling and dynamic index based diffusion. Opt. Lasers Eng..

[24] Gayathri J., Subashini S. (2018). A spatiotemporal chaotic image encryption scheme based on self adaptive model and dynamic keystream fetching technique. Multimed. Tools Appl..

[25] Wu X., Wang K., Wang X., Kan H., Kurths J. (2018). Color image DNA encryption using NCA map-based CML and one-time keys. Signal Process..

[26] Zhu C., Wang G., Sun K. (2018). Cryptanalysis and Improvement on an Image Encryption Algorithm Design Using a Novel Chaos Based S-Box. Symmetry.

[27] Zhu S., Wang G., Zhu C. (2019). A Secure and Fast Image Encryption Scheme Based on Double Chaotic S-Boxes. Entropy.

[28] Liu H., Zhao B., Huang L. (2019). Quantum image encryption scheme using Arnold transform and S-box scrambling. Entropy.

[29] Zhang Q., Guo L., Wei X. (2013). A novel image fusion encryption algorithm based on DNA sequence operation and hyper-chaotic system. Optik.

[30] Chai X., Chen Y., Broyde L. (2017). A novel chaos-based image encryption algorithm using DNA sequence operations. Opt. Lasers Eng..

[31] Chai X., Fu X., Gan Z., Lu Y., Chen Y. (2019). A color image cryptosystem based on dynamic DNA encryption and chaos. Signal Process..

[32] Khan J.S., Ahmad J., Abbasi S.F., Kayhan S.K. DNA Sequence Based Medical Image Encryption Scheme. Proceedings of the 10th Computer Science and Electronic Engineering (CEEC).

[33] Zhan K., Wei D., Shi J., Yu J. (2017). Cross-utilizing hyperchaotic and DNA sequences for image encryption. J. Electron. Imaging.

[34] Zhu C., Hu Y., Sun K. (2012). New image encryption algorithm based on hyperchaotic system and ciphertext diffusion in crisscross pattern. J. Electron. Inf. Tech..

[35] Sun S. (2018). A Novel Hyperchaotic Image Encryption Scheme Based on DNA Encoding, Pixel-Level Scrambling and Bit-Level Scrambling. IEEE Photonics J..

[36] Zhou N., Chen W., Yan X., Wang Y. (2018). Bit-level quantum color image encryption scheme with quantum cross-exchange operation and hyper-chaotic system. Quantum Inf. Process..

[37] Ahmed F., Siyal M., Abbas V. A perceptually scalable and jpeg compression tolerant image encryption scheme. Proceedings of the 4th Pacific-RIM Symposium on Image and Video Technology (PSIVT).

[38] Ahmad J., Khan M.A., Ahmed F., Khan J.S. (2018). A novel image encryption scheme based on orthogonal matrix, skew tent map, and XOR operation. Neural Comput. Appl..

[39] Hua Z., Zhou Y. (2017). Design of image cipher using block-based scrambling and image filtering. Inf. Sci..

[40] Rossler O.E. (1979). An equation for hyperchaos. Phys. Lett. A..

[41] Gu Q., Gao T. (2009). Analysis of transition between chaos and hyper-chaos of an improved hyper-chaotic system. Chin. Phys. B.

[42] Chen G., Ueta T. (1999). Yet another chaotic attractor. Int. J. Bifurc. Chaos.

[43] Adleman L.M. (1994). Molecular computation of solutions to combinatorial problems. Nature.

[44] Li T., Hu Z., Jia Y., Wu J., Zhou Y. (2018). Forecasting Crude Oil Prices Using Ensemble Empirical Mode Decomposition and Sparse Bayesian Learning. Energies.

[45] Zhao H., Zheng J., Xu J., Deng W. (2019). Fault diagnosis method based on principal component analysis and broad learning system. IEEE ACCESS.

[46] Li T., Zhou Y., Li X., Wu J., He T. (2019). Forecasting Daily Crude Oil Prices Using Improved CEEMDAN and Ridge Regression-Based Predictors. Energies.

[47] Alvarez G., Li S. (2006). Some basic cryptographic requirements for chaos-based cryptosystems. Int. J. Bifurc. Chaos.

[48] Yue W., Joseph P.N., Sos A. (2011). NPCR and UACI Randomness Tests for Image Encryption. J. Sel. Areas Telecommun..

[49] Chai X., Gan Z., Lu Y., Chen Y., Han D. (2017). A novel image encryption algorithm based on the chaotic system and DNA computing. Int. J. Mod. Phys. C.

